# Sustainable and resource-efficient synthesis of zeolite membranes in a fluoride-free, organic template-free dilute solution

**DOI:** 10.1039/d5ra03631c

**Published:** 2025-06-26

**Authors:** Liangqing Li, Jiajia Li, Yang Li, Xinyu Wu, Liangsong Li, Wenhao Zeng, Yajun Chen, Jungkyu Choi

**Affiliations:** a Key Laboratory of Functional Membranes and Energy Materials, School of Chemistry and Chemical Engineering, Huangshan University Huangshan 245041 China li_liangqing@126.com liliangqing@hsu.edu.cn; b School of Chemistry and Materials, University of Science and Technology of China Hefei 230026 China llq001@ustc.edu.cn; c Department of Chemical and Biological Engineering, Korea University Seoul 02841 Republic of Korea jungkyu_choi@korea.ac.kr; d Shanghai Branch, CNOOC Safety &Technology Services Limited Shanghai 200335 China; e Shanghai Safety and Environmental Protection Branch, CNOOC Energy Technology & Services Limited Shanghai 200335 China; f Liaoning Qingyang Chemical Industry Corporation Liaoyang 111000 China

## Abstract

Environmentally friendly and sustainable synthesis technologies hold considerable significance in the manufacturing of high-performance mordenite membranes. Herein, a high-performance mordenite membrane was successfully fabricated on a macroporous tube under fluoride-free and organic template-free conditions, using a highly diluted synthesis solution with a molar composition of H_2_O/SiO_2_ = 250. This approach eliminated the necessity of fluoride and template agents while reducing chemical usage *via* a highly diluted solution. The effects of the presence of seed layers and corresponding coating methods and crystallization temperature and time on membrane formation and isopropanol/water separation performance were systematically investigated. Results indicated that high-quality membrane formation was facilitated by a seed layer prepared *via* variable-temperature hot dip-coating on macroporous tubes. Under optimized preparation parameters, the resulting mordenite membrane achieved a permeation flux of 3.24 kg m^−2^ h^−1^ and a separation factor exceeding 10 000 in separation of isopropanol dehydration. These findings collectively demonstrate the potential of this fluoride-free, template-free, and diluted-solution approach for producing high-performance mordenite membranes boasting significant environmental and economic advantages.

## Abstract 2

A green and sustainable method was developed to prepare a high-performance mordenite membrane on a low-cost macroporous tube under fluoride-free and template-free conditions using a highly diluted synthesis solution for isopropanol dehydration.

## Graphical abstract

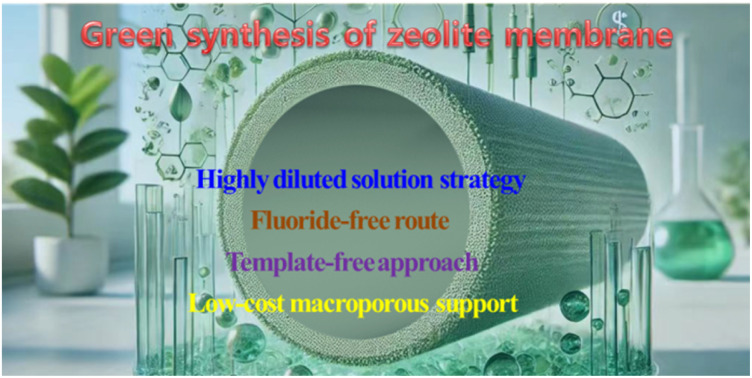

## 1 Introduction

Membrane-based separation technologies are extensively acknowledged for their compact design and energy efficiency compared to traditional, energy-demanding methods such as distillation, adsorption, and recrystallization.^1–3^ Considerable research has been conducted on various membrane materials, including zeolites,^4^ ceramics,^5^ and carbon molecular sieves,^6^ for applications in liquid separation, gas separation, and membrane reactor systems. Among these, zeolite membranes, characterized by their well-ordered molecular channels, appropriate pore sizes, as well as remarkable chemical and thermal stability, have demonstrated significant potential for liquid separation, even in extreme hydrothermal and chemical environments.^7^ Zeolite membranes exhibit optimal pore sizes and thus facilitate size-based selective separation of molecules, thereby enhancing separation efficiency.^8^ Over the past few decades, significant advancements have been achieved in advancing their preparation, characterization, and practical applications.^9–13^ Various fabrication techniques have been established to optimize the properties and performance of zeolite membranes, addressing both engineering challenges and economic concerns.^14–16^

Mordenite represents one of the earliest known zeolite molecular sieves, available in both natural and synthetic forms.^17^ It is extensively recognized for its medium silicon-to-aluminum ratio (typically in the range of 3–10), as well as its excellent thermal and mechanical stability.^18^ Mordenite belongs to the orthorhombic crystal system, featuring a pore structure composed primarily of twelve-membered and eight-membered ring channels. The twelve-membered ring windows are elliptical, exhibiting pore sizes of 6.5 × 7.0 Å^2^. Concurrently, the eight-membered ring channels feature dimensions of 2.6 × 5.7 Å^2^. Along the *c*-axis, straight channels are formed by both twelve-membered and eight-membered rings, with the latter situated between the former. In contrast, only eight-membered ring channels are present along the *b*-axis.^19^ Leveraging the unique properties of the mordenite zeolite, the resultant membrane will preserve many of its advantageous attributes. Indeed, mordenite membranes boast high hydrophilicity, which is directly linked to their relatively low silicon-to-aluminum ratio.^20^ Furthermore, the well-defined pore sizes of these membranes render them particularly effective in applications such as organic solvent dehydration, including isopropanol dehydration.^21^ These qualities collectively position mordenite membranes a promising material with potential for further development.^17–22^

Previous studies have devoted considerable endeavors to improving the quality of mordenite membranes. These efforts aim not only to enhance their performance but also to render the production processes more cost-efficient and environmentally sustainable.^14,15,18^ Early approaches to the preparation of mordenite membranes typically involve employing tetraethylammonium hydroxide (TEAOH)^23^ or tetraethylammonium bromide (TEABr)^24^ as organic template agents. However, upon hydrothermal crystallization, the organic template agents remain trapped within the pore structure of the membrane, obstructing the pores. High-temperature calcination is generally required to remove the templates. However, despite its excellent efficacy in removing the organic template agents, this process can still induce cracks and other defects in the original dense membrane, compromising the structural integrity and separation performance of zeolite membranes.^25^ Additionally, organic template agents are costly and are prone to thermal decomposition during subsequent high-temperature calcination. This decomposition releases toxic by-products that exacerbate environmental pollution.^25^ In recent years, researchers have explored methods for preparing mordenite membranes omitting the involvement of organic template agents.^26–34^ An organic template-free approach for synthesizing mordenite membranes on seeded tubular supports was first reported by Matsukata *et al.*, which showed a separation factor of 3360 and a flux of 0.1 kg m^−2^ h^−1^ for the dehydration of isopropyl alcohol *via* pervaporation, using a synthesis gel with a molar composition of H_2_O/SiO_2_ = 40.^29^ In a later work from the same group, a more concentrated synthesis gel with a H_2_O/SiO_2_ ratio of approximately 12.2 was used, which displayed an increased flux of 0.658 kg m^−2^ h^−1^ and a slightly higher separation factor of 4832.^30^ Subsequently, to further improve the isopropanol dehydration performance of mordenite membranes under organic template-free conditions, several studies explored the use of fluoride-containing media. Zhou *et al.* employed an NH_4_F-containing synthetic gel (H_2_O/SiO_2_ = 35) to prepare a mordenite membrane on a porous tubular mullite support, which resulted in a flux of 1.85 kg m^−2^ h^−1^ and a water/isopropyl alcohol selectivity of 3300.^31^ Zhu *et al.* synthesized mordenite membranes using a synthesis gel containing NaF with a H_2_O/SiO_2_ ration of 35 under microwave-assisted heating, which exhibited a permeation flux of 1.45 kg m^−2^ h^−1^ and a separation factor of high than 10 000.^32^ Gu *et al.* used a synthesis solution containing NH_4_F with a H_2_O/SiO_2_ ratio of 50 to fabricate mordenite membranes on four-channel Al_2_O_3_ hollow fiber substrates. The resulting membranes displayed a permeation flux of 1.43 kg m^−2^ h^−1^ and a separation selectivity exceeding 10 000.^33^ Recently, reported mordenite membranes synthesized *via* a novel intermittent hydrothermal method using a NaF-containing solution. The resulting membranes showed a permeation flux of 5.57 kg m^−2^ h^−1^ and a separation factor exceeding 10 000.^34^ These results indicate that fluoride-containing media, exemplified by NH_4_F and NaF, markedly improve the performance of mordenite membranes in organic template-free synthesis. Nevertheless, the incorporation of fluoride compounds also introduces several challenges. Fluoride materials pose a threat to the environment and make the handling and disposal processes more intricate.^35^ However, no high-performance mordenite membranes have yet been achieved for isopropanol dehydration under organic template-free and fluoride-free conditions, despite previous reports on such synthesis routes.

Herein, a high-performance mordenite membrane was achieved *via* a fluoride-free and organic template-free approach using a highly diluted synthesis solution with a molar composition of H_2_O/SiO_2_ = 250, on macroporous tubes. This work presents distinct advantages, including the use of a high H_2_O/SiO_2_ ratio, which enhances reagent utilization, improving material efficiency while reducing waste. By eliminating both fluoride and organic template agents, it not only lowers costs but also mitigates potential environmental concerns associated with their manufacturing. Meanwhile, satisfactory membrane performance is achieved. The findings presented in this paper provide a promising sustainable and resource-efficient route for the fabrication of high-performance mordenite membranes.

## 2 Experimental

### 2.1 Materials and reagents

A commercially available macroporous ceramic tube, composed primarily of α-alumina and characterized by a nominal average pore size of approximately 3 μm, was hereby used as a support. The tube has an inner diameter of 8 mm, an outer diameter of 12 mm, and a length of 50 mm. It was purchased with its outer surface already polished and cleaned, and utilized directly without additional treatment. Mordenite crystals, with a SiO_2_/Al_2_O_3_ molar ratio of 20 and sizes of 1 μm and 300 nm, were hereby used as seeds. Both the ceramic tubes and mordenite crystals were purchased from Hefei YuanFen New Materials Technology Co., Ltd; sodium hydroxide (NaOH, 96%), aluminum sulfate octadecahydrate (Al_2_(SO_4_)_3_·18H_2_O, 99%), and isopropanol ((CH_3_)_2_CHOH, 99.7%) were supplied by Sinopharm Chemical Reagent Co., Ltd; colloidal silica (25 wt% suspension in water) was obtained from Qingdao Haiwan Specialty Chemicals Co., Ltd, and deionized water (DI water) was prepared in-house.

### 2.2 Preparation of mordenite membranes

Both macroporous tubes without any seed layer and those with a seeded coating layer were utilized for the preparation of mordenite membranes. The seed tubes were prepared by coating the outer surface of macroporous tubes with mordenite crystals using the previously reported hot dip-coating method (HD) or temperature-varying hot dip-coating method (VTHD).^18,36^ For the HD method, the support was pre-heated to 80 °C and then dipped into a 0.2 wt% aqueous suspension of 1 μm-sized mordenite crystals for 20 s, followed by drying at 80 °C for 3 h. For the VTHD method, the procedure consisted of three steps: (1) the support was pre-heated to 175 °C and dipped into a 2.0 wt% aqueous suspension of 1 μm-sized mordenite crystals for 20 s, followed by drying at 80 °C for 3 h; (2) the outer surface of the dried support was gently rubbed using absorbent cotton to remove excess and loosely attached crystals until no visible particles remained; (3) the support was then pre-heated to 80 °C and dipped into a 0.2 wt% aqueous suspension of 300 nm-sized mordenite crystals for 20 s, followed by drying at 80 °C for 3 h. The seeded supports obtained by these two methods were subsequently used for membrane preparation. The membrane synthesis solution for the mordenite membrane was prepared by mixing the colloidal silica, NaOH, Al_2_(SO_4_)_3_·18H_2_O, and DI water: the molar composition of SiO_2_, Na_2_O, Al_2_O_3_, and H_2_O was 1 : 0.26 : 0.06 : 250. Compared to the conventional synthesis solutions or gels, which typically have H_2_O/SiO_2_ ratios in the range of approximately 12.2 to 50,^29–33^ the solution used in this study was more diluted, with a H_2_O/SiO_2_ ratio of 250. Upon thoroughly stirring the solution, it was transferred into a stainless steel autoclave, along with the tubes (non-seeded or seeded), and placed in an oven for the crystallization reaction. Following crystallization completion, the autoclave was removed from the oven and quenched using tap water. The membrane tubes were subsequently recovered from the autoclave and washed with DI water thoroughly until the solution became nearly neutral. After that, the tubes were placed in an oven and dried at 120 °C overnight. Membrane tubes (referred to as MT01 to MT10) were fabricated following this procedure. The specific preparation parameters are listed in Table 1.

**Table 1:** Preparation parameters of MT01–MT10 membranes

No.	Synthesis conditions		
	Seed layer preparation	Crystallization temperature (°C)	Crystallization time (h)
MT01	—a	165	24
MT02	HD^b^	165	24
MT03	VTHD^c^	165	24
MT04	VTHD	150	24
MT05	VTHD	160	24
MT06	VTHD	170	24
MT07	VTHD	180	24
MT08	VTHD	165	12
MT09	VTHD	165	36
MT10	VTHD	165	48

a No seed layer was prepared.

b Seed layer was prepared using the hot dip-coating method.

c Seed layer was prepared using the temperature-varying hot dip-coating method.

### 2.3 Characterization

Herein, the fabricated membrane samples were characterized by scanning electron microscopy (SEM) and energy-dispersive X-ray spectroscopy (EDS) using a Hitachi SU8600 instrument (Japan) and X-ray diffraction (XRD) using a Rigaku SmartLab 9 kW analytical X-ray diffractometer (Japan) with conventional Cu Kα radiation. The SEM images were obtained following gold sputtering, with an accelerating voltage range of 5–20 kV. The single-component gas permeation test was conducted to evaluate membrane compactness. The experiment was carried out at room temperature using hydrogen (H_2_), nitrogen (N_2_), and sulfur hexafluoride (SF_6_) gases, each with a purity of 99.999%. The feed side was supplied with the test gas at a constant pressure of 0.1 MPa, while the permeate side was kept at atmospheric pressure. The pervaporation performance of the membrane was assessed by dehydrating a isopropanol/water (90 : 10 by mass) mixture at 75 °C using a custom-built laboratory apparatus. Permeation flux and separation factor were determined using the method described in ref. 34 The compositions of the feed and permeate samples were analyzed using gas chromatography (GC) equipped with a thermal conductivity detector (TCD) and a Shimadzu GC-2018 (Japan).

## 3 Results and discussion

### 3.1 Preparation of mordenite membrane on the macroporous ceramic tube

**Fig. 1:**
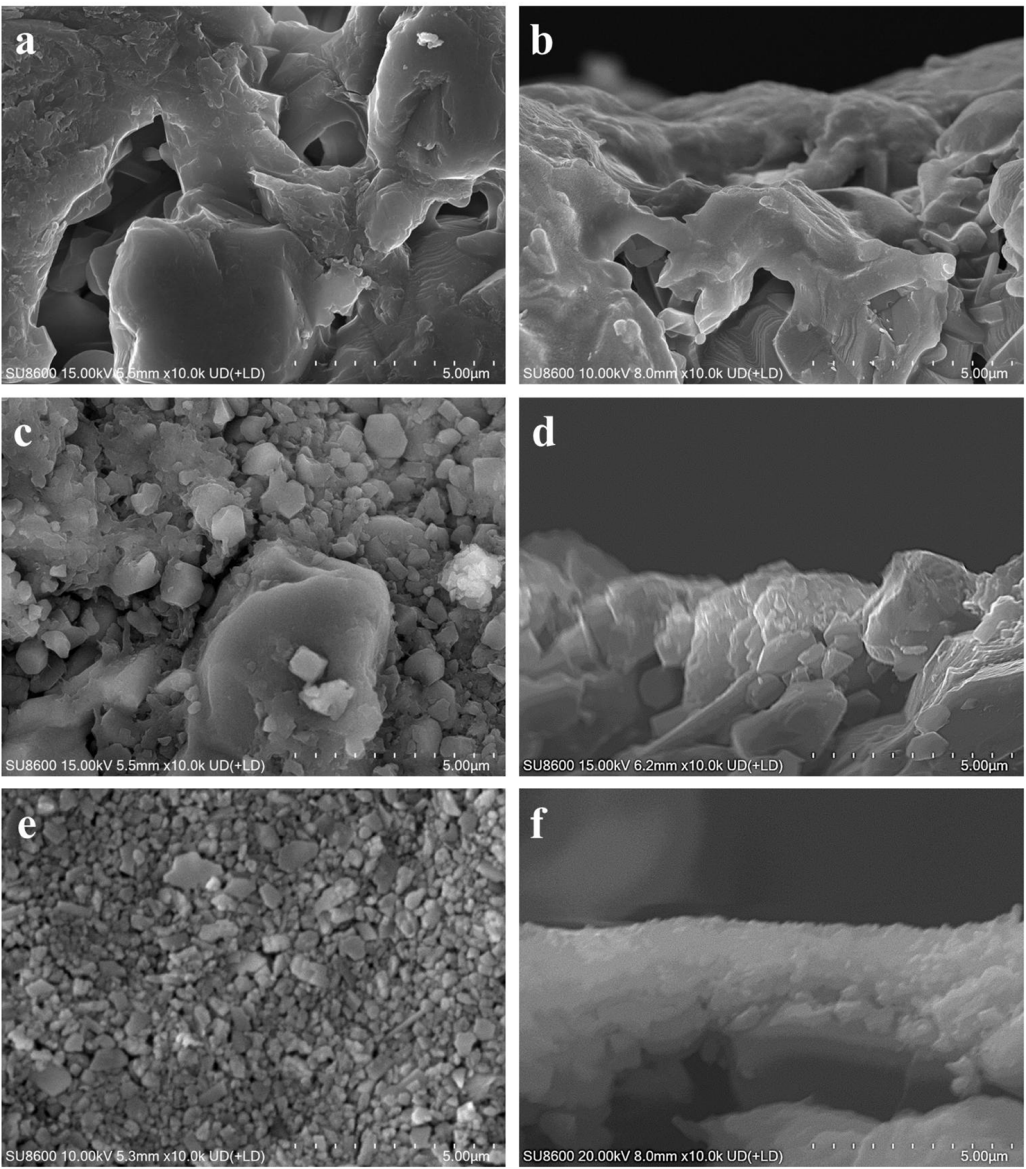
Surface and cross-sectional SEM images of (a and b) the original macroporous ceramic tube without a seed layer, (c and d) the tube seeded using the hot dip-coating method, and (e and f) the tube seeded using the temperature-varying hot dip-coating method.

This study delved into the synthesis of mordenite membranes within a dilute solution that lacked both fluoride and organic templates. Its objective was to tackle the environmental concerns stemming from chemical usage and to offer a sustainable and resource-efficient methodology for the process. Building upon this approach, the impact of the seed layer on membrane formation was initially examined by comparing membranes prepared on the non-seeded and seeded tubes. The macroporous ceramic tube, which was made up of a multitude of α-alumina grains with diverse and irregular shapes, was formed through high-pressure assembly. It featured a wide range of sizes in the interparticle pores within it. The original support (*i.e.*, without a seed layer) exhibited randomly distributed pores, some with surface openings exceeding 5 μm, as shown in Fig. 1a and b. The macroporous support possessed a highly porous structure, providing a surface for zeolite crystal growth. Given the continuous zeolite membrane formation, this support could provide high mechanical and thermal stabilities, crucial for robust long-term uses. To draw a comparison, a seed layer was hereby formed on the macroporous tube surface using the hot dip-coating method and the temperature-varying hot dip-coating method, followed by the subsequent hydrothermal growth for the preparation of mordenite membranes. SEM images of the supports with the seed layer are shown in Fig. 1c–f. Following seed deposition, the surface pore size and roughness of the support decreased, compared to the original support without the seed layer (Fig. 1a). In particular, using the varying temperature hot dip-coating method, the support surface was completely covered by the seed layer, with no α-alumina grains being exposed (Fig. 1e). Conversely, employing the hot dip-coating method resulted in the filling of surface voids in the support with numerous crystal particles, yet some α-alumina grains were still exposed (Fig. 1c). The comparison of cross-sectional SEM images of the three different supports (Fig. 1b, d and f) revealed that the formed seed layer rendered the support surface significantly flattened when using the temperature-varying hot dip-coating method. These supports were subsequently used to prepare mordenite membranes in a fluoride-free, organic template-free dilute synthesis solution at a crystallization temperature of 165 °C for 24 h.

**Fig. 2:**
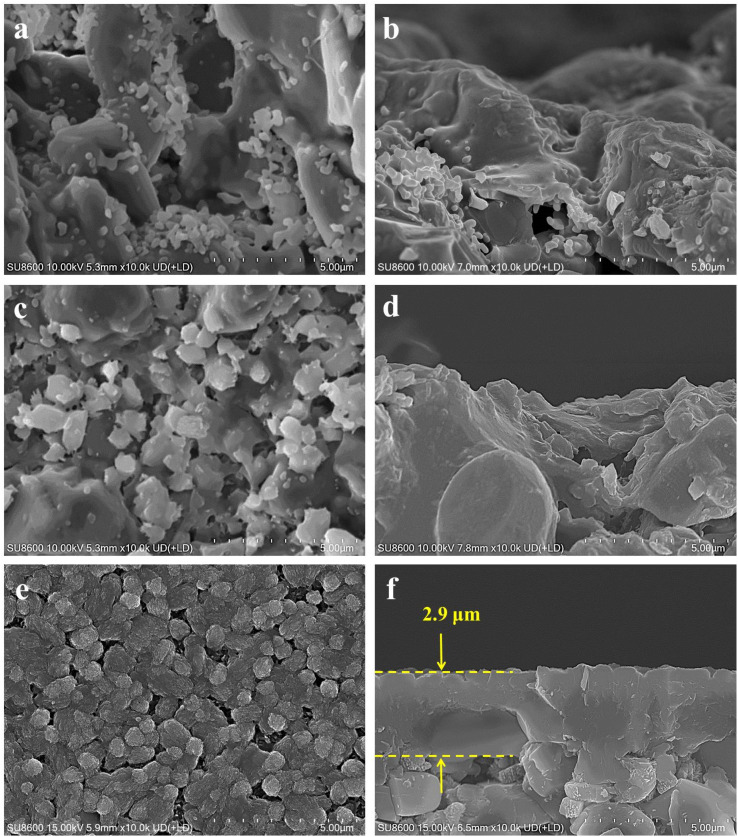
Surface and cross-sectional SEM images of membranes (MT01, MT02, and MT03) prepared on (a and b) the original macroporous ceramic tube without a seed layer, (c and d) the tube seeded using the hot dip-coating method, and (e and f) the tube seeded using the temp erature-varying hot dip-coating method.

Fig. 2 presents the morphological difference of the mordenite membranes (MT01, MT02, and MT03) prepared on different supports shown in Fig. 1. Fig. 2a and b illustrate the morphology of membrane MT01 directly prepared on the original macroporous tube without a seed layer. A large number of sub-micron particles existed in an aggregated state and stuck to the surface of the α-alumina grains. They partially filled and covered the surface voids. As a result, the pores of the support were still distinctly open and observable, and there was no continuous membrane layer to be seen on the surface of the support. EDS analysis revealed an extremely low Si/Al ratio of 0.13 on the surface of membrane MT01, attributable to incomplete formation of a continuous membrane layer and the primary contribution of the α-alumina support. Consistently, the membrane MT01 exhibited extremely poor pervaporation performance when dealing with the isopropanol/water mixture. Specifically, its separation factor was found to be lower than 2 (Table 2). This result aligned with the SEM observations. Fig. 2c and d show the morphology of membrane MT02, prepared on a support with a seed layer formed using the hot dip-coating method. Compared to membrane MT01, the particles within the voids of the support surface in membrane MT02 were larger and exhibited a greater coverage. EDS analysis revealed a Si/Al ratio of 0.46 on the surface of membrane MT02, showing a slight increase compared to MT01. This increase was ascribable to the enhanced particle formation on the membrane surface, apparently due to the presence of coated mordenite seeds on the support. This, in turn, diminished the contribution of aluminum from the support to the EDS results. As expected from the incomplete filling of the surface void, membrane MT02 exhibited poor separation performance when handling the isopropanol/water mixture. Similar to membrane MT01, it had virtually no separation selectivity. The sole distinction was that its permeation flux had decreased slightly (Table 2). This suggested that while the seed layer formation by the hot dip-coating method improved the membrane layer's density, its degree was not sufficient to achieve effective separation of the isopropanol/water mixture. The morphology of membrane MT03, prepared using a support with a seed layer formed by the temperature-varying hot dip-coating method, is shown in Fig. 2e and f. Notably, the support surface was completely covered by numerous large and inter-grown particles. Its morphology was distinctly different from that of membranes MT01 and MT02. Furthermore, the cross-sectional image in Fig. 2f showed a dense membrane layer formed on the support surface, with a thickness of approximately 2.9 μm. In this work, the single-component gas permeances of a membrane prepared under the same conditions as MT03 for H_2_, N_2_, and SF_6_ were 1.78, 0.62, and 0.16 × 10^−8^ mol m^−2^ s^−1^ Pa^−1^, respectively, with ideal selectivities of H_2_/N_2_ and H_2_/SF_6_ of 2.9 and 11.1. These values are comparable to those reported in the previous study employing the intermittent heating method and are higher than those obtained using conventional heating in the same study.^34^ Moreover, the ideal selectivity of H_2_/SF_6_ exceeds the corresponding Knudsen selectivity (8.5), further indicating the good compactness of the membrane synthesized under the same conditions as those used for MT03.

**Table 2:** Membrane parameters and separation performance of MT01–MT10 for the isopropanol/water (90 : 10 by mass) mixture at 75 °C

No.	Membrane parameters		Separation performances	
	Thickness (μm)	Si/Al_(EDS)_^a^	Flux (kg m^−2^ h^−1^)	S.F.
MT01	—b	0.13	>200	<2
MT02	—	0.46	178.92	< 2
MT03	2.9	3.35	3.24	>10 000
MT04	1.6	2.08	85.68	<2
MT05	2.0	2.17	11.24	6
MT06	3.5	3.48	2.77	>10 000
MT07	3.9	5.45	1.93	>10 000
MT08	1.4	1.71	155.41	<2
MT09	4.4	3.50	2.41	>10 000
MT10	5.2	3.69	1.80	>10 000

a The Si/Al_(EDS)_ ratio was determined by EDS analysis of the silicon (Si) and aluminum (Al) elements on the sample membrane surface.

b No obvious continuous membrane layer was observed.

**Fig. 3:**
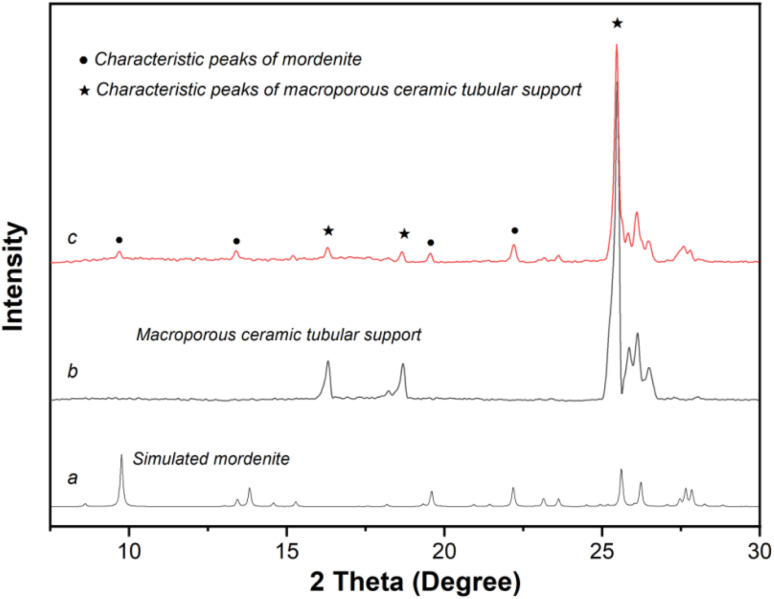
XRD patterns of (a) simulated mordenite crystals, (b) macroporous ceramic tube, and (c) membrane MT03 prepared on the tube seeded using the temperature-varying hot dip-coating method.

Fig. 3 displays the XRD patterns of the original macroporous ceramic tube (Fig. 3b), simulated mordenite crystals (Fig. 3a), and membrane MT03 (Fig. 3c). Evidently, distinct characteristic peaks of the macroporous tube support and mordenite crystalline phase were observed. A combination of the XRD and SEM analyses confirmed the successful preparation of a continuous mordenite membrane on the support surface (*i.e.*, membrane MT03), for which the seed layer had been formed using the temperature-varying hot dip-coating method. The Si/Al ratio on the surface of membrane MT03, as determined by EDS analysis, was *ca.* 3.35, significantly higher than that of membranes MT01 and MT02. This elevated Si/Al ratio was primarily attributed to the formation of a continuous membrane layer, approximately 2.9 μm thick, on the macroporous tube support surface. This significantly minimized the contribution of α-alumina from the support to the EDS results. Furthermore, the continuity of the mordenite membrane of membrane MT03 resulted in a permeation flux of 3.24 kg m^−2^ h^−1^ and a separation factor exceeding 10 000 for the dehydration of isopropanol/water mixture (Table 2).

Collectively, these results demonstrated that the application of the temperature-varying hot dip-coating technique to create a seed layer on the outer surface of the macroporous tube enabled the successful production of a continuous and compact mordenite membrane. This achievement was realized under conditions characterized by the absence of fluoride, the lack of a template, and the use of a dilute solution. Desirably, this membrane exhibited excellent separation performance for isopropanol/water mixture dehydration. Typically, the seed layer is critical for membrane formation, as it reduces the pore size and surface roughness of the support while providing nucleation sites for zeolite crystal growth, thereby determining the final membrane quality.^36^ In particular, when it comes to creating a uniform and flawless seed layer on macroporous supports, it poses a major hurdle. This is due to the relatively large pore sizes of these supports, which heighten the likelihood of defects occurring in the final membrane.^37^ Nevertheless, cheap macroporous ceramic tubes remain attractive for industrial applications because of their substantially lower cost. Notably, the ceramic support typically accounts for at least 70% of the total cost of a zeolite membrane, rendering the use of inexpensive macroporous supports crucial for economic feasibility.^38^ In this study, the proposed approach proved to be efficient in manufacturing mordenite membrane on macroporous tubes. It accomplished this by yielding superior seed coverage and facilitating the formation of a dense membrane layer. Moreover, the secondary growth condition, which allowed for the use of a fluoride-free, template-free, and dilute solution, provided both environmental and economic advantages. These benefits were realized by reducing hazardous chemicals, simplifying the synthesis process, and lowering material costs.

### 3.2 Effect of crystallization temperature

Crystallization temperature constitutes a key factor affecting zeolite membrane formation by directly modulating the crystallization kinetics of the zeolite phase.^39^ Accordingly, temperature variations impact the nucleation and growth rates of zeolite crystals, thereby affecting the membrane's morphology and continuity. This, in turn, determines the overall membrane quality and separation performance. In this section, the influence of crystallization temperature on the microstructure and separation performance of the zeolite membranes was examined and addressed using the complementary characterizations (SEM, XRD, and EDS) and pervaporation performance tests.

**Fig. 4:**
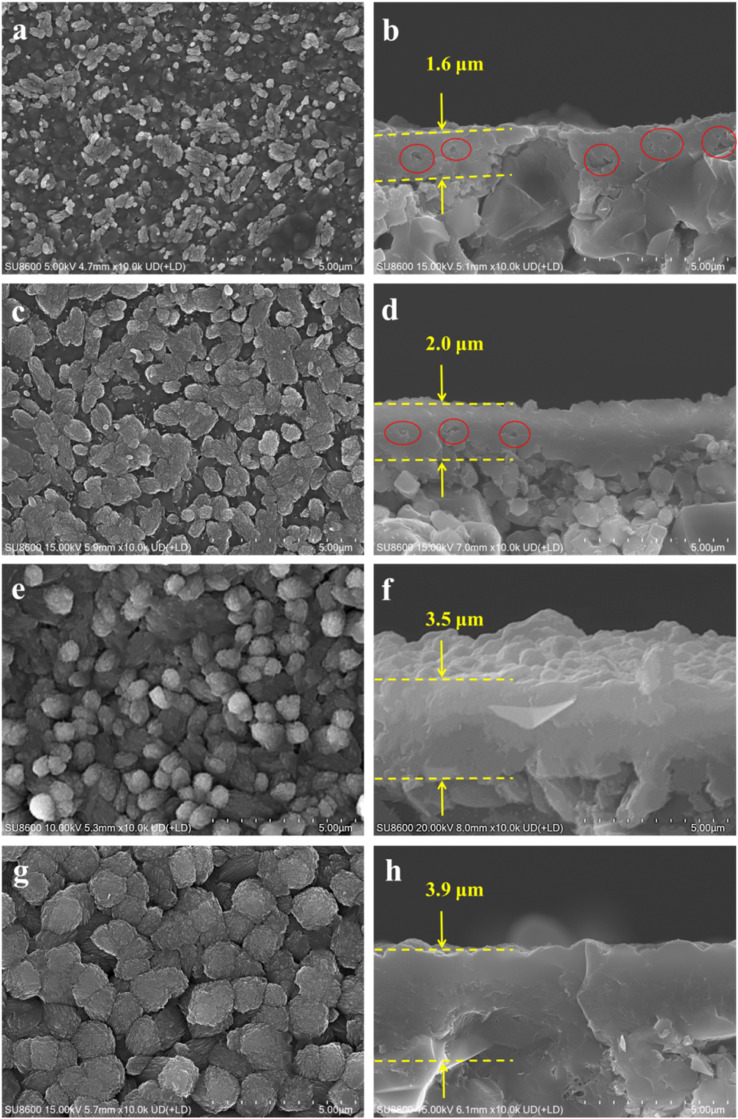
Surface and cross-sectional SEM images of membranes (MT04, MT05, MT06, and MT07) prepared at crystallization temperatures of (a and b) 150 °C, (c and d) 160 °C, (e and f) 170 °C, and (g and h) 180 °C.

**Fig. 5:**
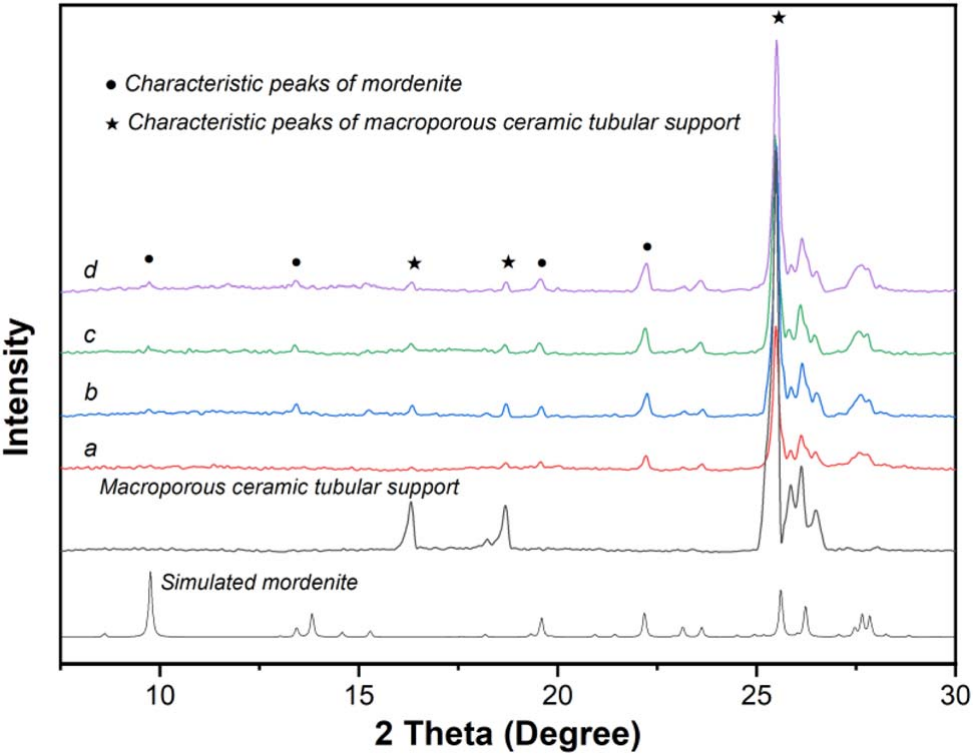
XRD patterns of membranes (MT04, MT05, MT06, and MT07) prepared at crystallization temperatures of (a) 150 °C, (b) 160 °C, (c) 170 °C, and (d) 180 °C.

Membrane MT03, synthesized at 165 °C, served as a benchmark for evaluating the effect of crystallization temperature on membrane formation. Membranes MT04, MT05, MT06, and MT07 underwent crystallization at temperatures of 150, 160, 170, and 180 °C, respectively. Their SEM images and XRD patterns are shown in Fig. 4 and 5, respectively. For membrane MT04 synthesized at 150 °C, a gel-like layer was observed covering the support surface, with randomly dispersed particles forming on its surface (Fig. 4a). The cross-sectional SEM image of MT04 revealed a relatively thin membrane layer of approximately 1.6 μm and relatively large pores within the membrane layer, as indicated by red circles (Fig. 4b). Seemingly, the large α-alumina grains did not achieve complete integration or interlocking with the membrane layer. When the crystallization temperature was set at 160 °C, membrane MT05 exhibited larger particles on its surface compared to membrane MT04 (Fig. 4c). Some of these particles were inter-grown, and there was an increase in the membrane thickness to roughly 2.0 μm. While the relatively large pores were still observed in the cross-sectional image of the membrane layer of MT05 (indicated by red circles in Fig. 4d), their number was reduced compared to MT04. Contrasting with MT05, the surface particles of the reference sample (membrane MT03) became more densely packed, and the cross-sectional image revealed a well-compacted membrane layer with no apparent pores inside. A dense membrane structure with a thickness of approximately 2.9 μm was thus formed (Fig. 2e and f). At a synthesis temperature of 170 °C, numerous particles densely covered the surface of membrane MT06, with a corresponding membrane thickness of approximately 3.5 μm (Fig. 4e and f). Upon increasing the synthesis temperature to 180 °C, the surface particles of membrane MT07 were markedly larger than those of MT06. Moreover, the membrane thickness further increased to approximately 3.9 μm (Fig. 4g and h). The densely packed particles formed a compact membrane layer on the support surface, while the membrane growth was further extended into the underlying pores beneath the membrane layer. These particles were closely integrated with the membrane layer, establishing a nearly continuous structure. However, filling the support pores with newly-formed particles beneath the main membrane layer would increase mass transfer resistance, thereby negatively impacting the membrane separation performance.

The XRD patterns of membranes MT04, MT05, MT06, and MT07 (Fig. 5) all exhibited characteristic peaks of mordenite and the macroporous tube support. This confirmed the successful formation of mordenite membranes on the macroporous tube under varying crystallization temperatures, corroborated by the SEM images displaying the membrane morphology. By examining the strongest mordenite peak at 2*θ* = 22°–23°, it was observed that its intensity gradually increased as the crystallization temperature rose from 150 to 180 °C. This indicated that higher crystallization temperatures promoted membrane growth. Consistently, the Si/Al ratio on the membrane surface increased gradually as the synthesis temperature rose from 150 to 180 °C: the corresponding values obtained from the EDS measurement were approximately 2.08, 2.17, 3.35, 3.48, and 5.45, respectively. The permeation fluxes of the membranes synthesized at 150, 160, 165, 170, and 180 °C (*i.e.*, MT04, MT05, MT03, MT06, and MT07) were 85.68, 11.24, 3.24, 2.77, and 1.93 kg m^−2^ h^−1^, respectively. Concurrently, the corresponding separation factors were <2, 6, >10 000, >10 000, and >10 000, respectively. This trend was primarily attributed to the coupled effects of membrane compactness and membrane thickness. For membranes synthesized at 165, 170, and 180 °C, the permeation fluxes of MT06 and MT07 were lower than that of MT03. This followed a general trend of decreasing flux with increasing synthesis temperature. This reduction primarily arose from the combined effects of increased membrane thickness (on top) and the progressive formation/growth of the membrane layer within the support pores in the deeper region (below the main membrane layer). The progressive particle formation and growth within the support pores in the deeper regions exacerbated resistance to molecular transport during separation, culminating in a substantial reduction in the permeation flux.

Optimizing the synthesis temperature holds considerable significance for striking a balance between membrane thickness (mainly relevant to the permeation flux) and compactness (mainly relevant to the separation factor) in isopropanol/water separation. Among the tested membranes, MT03 delivered the best pervaporation performance, yielding a permeation flux of 3.24 kg m^−2^ h^−1^ and a separation factor exceeding 10 000. This finding demonstrated the synthesis temperature of 165 °C as the optimal value for fabricating high-quality mordenite membranes on macroporous tubes under fluoride-free, template-free, and diluted solution conditions in the present work.

### 3.3 Effect of crystallization time

**Fig. 6:**
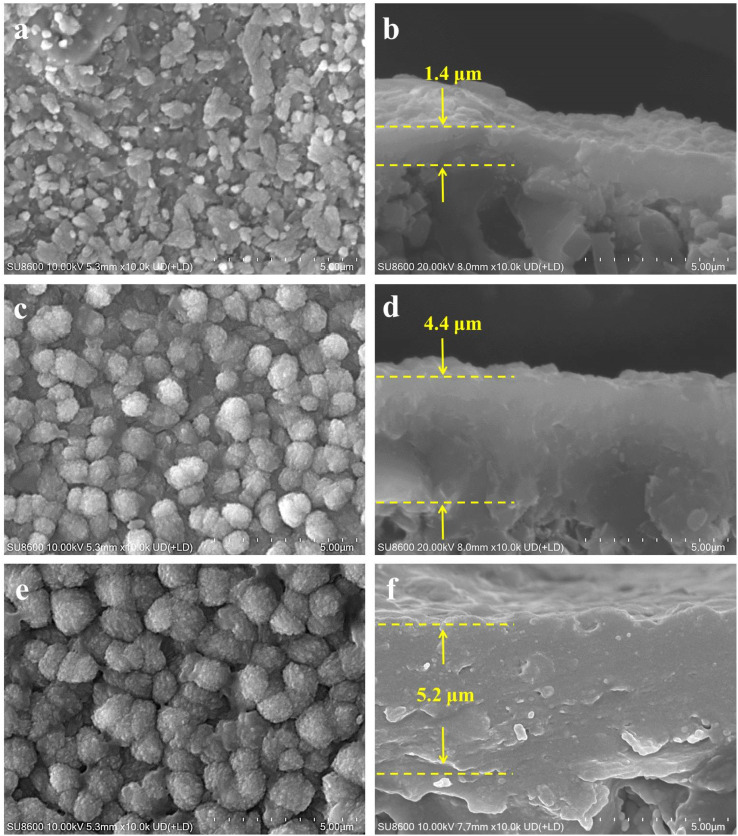
Surface and cross-sectional SEM images of membranes (MT08, MT09, and MT10) prepared at crystallization times of (a and b) 12 h, (c and d) 36 h, and (e and f) 48 h.

**Fig. 7:**
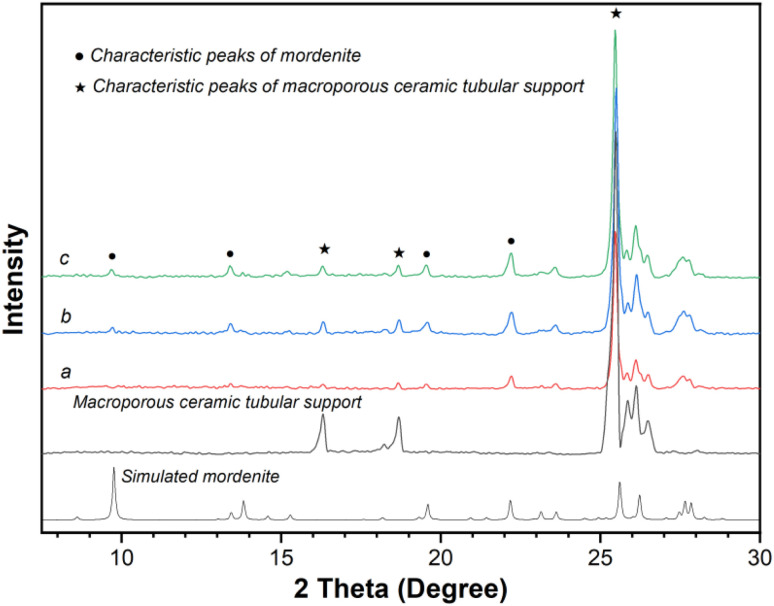
XRD patterns of membranes (MT08, MT09, and MT10) prepared at crystallization times of (a) 12 h, (b) 36 h, and (c) 48 h.

In addition to the synthesis temperature, zeolite membranes are composed of intergrown crystals and are likely to exhibit altered properties as the crystallization time varies.^40^ To systematically investigate this effect, membrane MT03 synthesized with a crystallization time of 24 h was hereby selected as the reference. Additional membranes were prepared with crystallization times of 12, 36, and 48 h to examine the effects of varying durations on membrane microstructure, morphology, and separation performance. The morphologies and microstructures of the membranes were analyzed using SEM and XRD, with the corresponding results presented in Fig. 6 and 7, respectively. At a crystallization time of 12 h, the surface of membrane MT08 was covered with particles and featured observable pinholes (Fig. 6a). The cross-sectional view revealed a thickness of approximately 1.4 μm of the membrane layer (Fig. 6b). As the crystallization time was extended to 24 h, larger particles appeared on the surface of the reference membrane MT03. Meanwhile, the corresponding membrane thickness increased to approximately 2.9 μm (Fig. 2e and f). As the synthesis time was further extended to 36 and 48 h, membranes MT09 and MT10 were densely covered with particles, and the particle size increased with prolonged synthesis time (Fig. 6c and e). The membrane thickness of MT09 was measured to be 4.4 μm, while that of MT10 was 5.2 μm. In both cases, a large number of particles formed within the support structure. These particles integrated with the α-alumina grains and became part of the additional membrane layer.

The XRD patterns of membranes MT08, MT09, and MT10 (Fig. 7) exhibited typical characteristic peaks of mordenite zeolite crystals and the macroporous tube support. The XRD analysis, which was carried out based on the peak intensities at 2*θ* = 22°–23°, revealed that when the crystallization time was longer, stronger peaks were obtained. This result indicated that an extended crystallization time contributed to the growth of the membrane layer. As the crystallization time was extended from 12 through 24 and 36 to 48 h, the Si/Al ratio on the membrane surface monotonically increased from 1.71 through 3.35 and 3.50 to 3.69, respectively (Table 2). At a low crystallization time of 12 h, the low Si/Al ratio on the membrane surface was attributable to the thin membrane layer.

As shown in Table 2, at a low crystallization time of 12 h, membrane MT08 exhibited almost no separation selectivity for isopropanol dehydration, apparently owing to the insufficient crystallization. When the crystallization time increased to 24 h, the reference membrane MT03 demonstrated excellent isopropanol dehydration selectivity, involving a separation factor exceeding 10 000 and a corresponding permeation flux of 3.24 kg m^−2^ h^−1^. Further extending the crystallization time to 36 and 48 h resulted in the increased separation selectivity, with both membranes showing the separation factors above 10 000. However, compared to membrane MT03, their permeation flux decreased monotonically to 2.41 and 1.80 kg m^−2^ h^−1^. This was apparently due to the increased membrane thickness with time, consistent with the thickness characteristics shown in Fig. 6d and f.

These results jointly demonstrated crystallization time as a critical parameter in preparing mordenite membranes, directly affecting membrane density and thickness. Appropriately extending the crystallization time could facilitate the formation of dense mordenite membranes. At the optimized crystallization time of 24 h, a high-quality mordenite membrane with a thin layer was successfully fabricated on the macroporous tube. This membrane demonstrated excellent separation performance for dehydration of isopropanol/water mixture.

### 3.4 Separation performance comparison

**Fig. 8:**
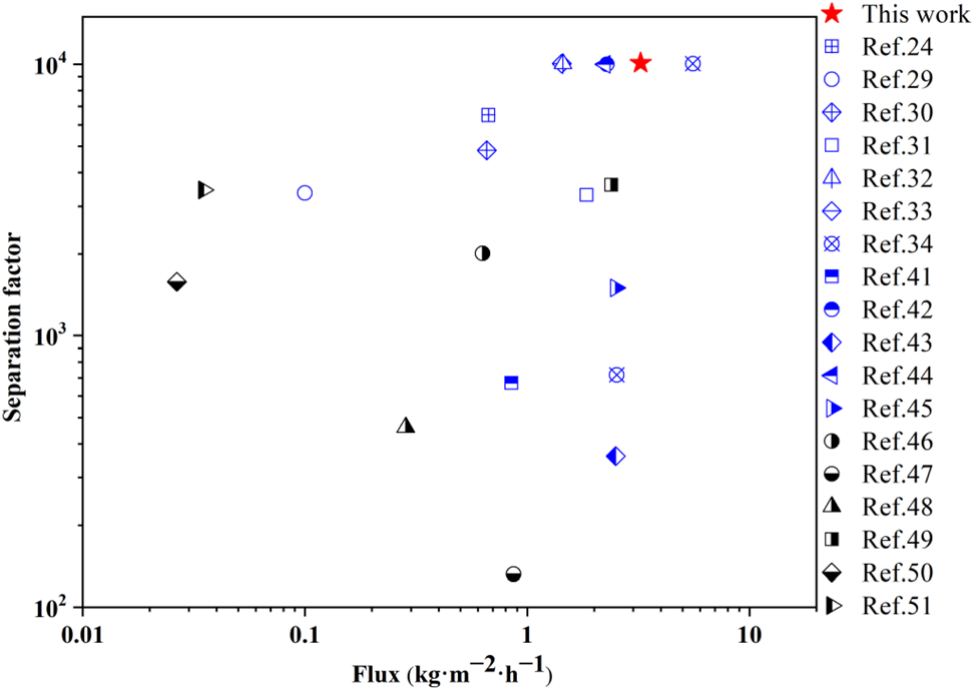
Pervaporation performance of membrane MT03 in flux *vs.* water/isopropanol selectivity plot in comparison with that of other membranes.

In order to conduct a more in-depth assessment of the separation performance of the mordenite membranes, membrane MT03 was selected. This membrane had exhibited the most superior performance among all the samples that were prepared during the course of this study. Its isopropanol dehydration performance was compared with the previously reported performances of mordenite membranes, other types of zeolite membranes, and other types of materials (Fig. 8 and Table 3).^24,29–34,41–50^ As shown in Table 3, many zeolite membranes, including MT03, exhibit generally better separation performance than most other types of membrane materials for isopropanol dehydration. Notably, the mordenite membrane previously synthesized by Li *et al.* using a fluoride-containing system and an intermittent heating method exhibited the best separation performance, achieving a permeation flux of up to 5.57 kg m^−2^ h^−1^ and a separation factor exceeding 10 000.^34^ This high flux was attributed to the reduced membrane thickness of approximately 1.9 μm achieved *via* the intermittent heating method, compared to about 4.3 μm using conventional heating in the same study, which necessitated the use of fluoride during synthesis. Correspondingly, the permeation flux increased from 2.53 to 5.57 kg m^−2^ h^−1^.^34^ Although the membrane synthesized *via* the intermittent heating method exhibited excellent separation performance, this approach might be too difficult for regulating the synthesis temperature in a stainless steel autoclave through desired natural cooling and heating within a given time in large-scale synthesis processes for industrial application. In contrast, membrane MT03 prepared in this work exhibited a slightly lower yet still remarkable isopropanol dehydration performance, achieving a permeation flux of 3.24 kg m^−2^ h^−1^ and a separation factor greater than 10 000, the highest among all reported membranes, excluding those synthesized by the intermittent heating method using fluoride-containing systems. Moreover, this study successfully manufactured high-performance mordenite membranes through a fluoride-free and template-free synthesis using a highly diluted solution, underscoring the significance of this work in developing and advancing a more environmentally friendly and efficient approach for mordenite membrane fabrication.

**Table 3:** Comparison of separation performance of various membranes for isopropanol dehydration by pervaporation or vapor permeation

Membrane materials	Isopropanol/water ratio (by mass) in feed	Separation temperature (°C)	Sep. performance		References
			Flux (kg m^−2^ h^−1^)	S.F.	
Mordenite	90 : 10	75	3.24	>10 000	This work
Mordenite	85 : 15	70	0.67	6500	24
Mordenite	90 : 10	75	0.10	3360	29
Mordenite	90 : 10	75	0.658	4832	30
Mordenite	90 : 10	75	1.85	3300	31
Mordenite	90 : 10	75	1.45	>10 000	32
Mordenite	90 : 10	75	1.43	>10 000	33
Mordenite	90 : 10	75	5.57	>10 000	34
Mordenite	90 : 10	75	2.53	718	34
ZSM-5	90 : 10	75	0.85	670	41
NaA	90 : 10	75	2.28	10 000	42
NaY	90 : 10	75	2.5	360	43
Zeolite T	90 : 10	75	2.24	10 000	44
CHA	90 : 10	105	2.5	1500	45
PVA/ZSM-5	90 : 10	60	0.63	2013	46
PVA/ZIF-8	90 : 10	30	0.868	132	47
CS-TEOS	90 : 10	80	0.284	460	48
GO/PAT	90 : 10	70	2.39	3600	49
PVA/silicone	90 : 10	30	0.0265	1580	50
SPVA	90 : 10	40	0.035	3452	51

## 4 Conclusions

In the current study, an environmentally friendly and sustainable technology was reported for fabricating mordenite membranes, which were successfully prepared on macroporous tubes using a fluoride-free, template-free approach involving a highly diluted synthesis solution (H_2_O/SiO_2_ = 250). The current approach addressed environmental concerns and reduces both synthesis costs and chemical consumption by eliminating the necessity for fluoride and organic templates. The systematic investigation of key synthesis parameters, such as the presence of seed layer and its formation and crystallization temperature and time, revealed their significant impact on membrane formation and separation performance. Under optimized conditions, the resulting mordenite membranes exhibited a permeation flux of 3.24 kg m^−2^ h^−1^ and a separation factor exceeding 10 000 for isopropanol dehydration. Overall, this work presents a promising, eco-friendly solution for synthesizing high-performance mordenite membranes. It offers a sustainable and economical approach that can be applied for the future production of these membranes on an industrial scale.

## Author contributions

Liangqing Li: conceptualization, methodology, investigation, data analysis, writing – original draft, writing – review and editing, and funding acquisition. Jiajia Li: conceptualization, methodology, data analysis, and writing – review and editing. Liangqing Li and Jiajia Li contributed equally to this work. Yang Li and Xinyu Wu: investigation, and validation. Liangsong Li, Wenhao Zeng, and Yajun Chen: investigation. Jungkyu Choi: writing – review and editing.

## Conflicts of interest

There are no conflicts to declare.

## Data Availability

All data supporting the findings of this study are available within the article.
